# Hybrid Incompatibility of the Plant Immune System: An Opposite Force to Heterosis Equilibrating Hybrid Performances

**DOI:** 10.3389/fpls.2020.576796

**Published:** 2021-02-16

**Authors:** Vanesa Calvo-Baltanás, Jinge Wang, Eunyoung Chae

**Affiliations:** Department of Biological Sciences, National University of Singapore, Singapore, Singapore

**Keywords:** hybrid necrosis, autoimmunity, anti-hybrid necrosis, heterosis, immunity, trade-off, NLR, growth

## Abstract

Hybridization is a core element in modern rice breeding as beneficial combinations of two parental genomes often result in the expression of heterosis. On the contrary, genetic incompatibility between parents can manifest as hybrid necrosis, which leads to tissue necrosis accompanied by compromised growth and/or reduced reproductive success. Genetic and molecular studies of hybrid necrosis in numerous plant species revealed that such self-destructing symptoms in most cases are attributed to autoimmunity: plant immune responses are inadvertently activated in the absence of pathogenic invasion. Autoimmunity in hybrids predominantly occurs due to a conflict involving a member of the major plant immune receptor family, the nucleotide-binding domain and leucine-rich repeat containing protein (NLR; formerly known as NBS-LRR). NLR genes are associated with disease resistance traits, and recent population datasets reveal tremendous diversity in this class of immune receptors. Cases of hybrid necrosis involving highly polymorphic NLRs as major causes suggest that diversified *R* gene repertoires found in different lineages would require a compatible immune match for hybridization, which is a prerequisite to ensure increased fitness in the resulting hybrids. In this review, we overview recent genetic and molecular findings on hybrid necrosis in multiple plant species to provide an insight on how the trade-off between growth and immunity is equilibrated to affect hybrid performances. We also revisit the cases of hybrid weakness in which immune system components are found or implicated to play a causative role. Based on our understanding on the trade-off, we propose that the immune system incompatibility in plants might play an opposite force to restrict the expression of heterosis in hybrids. The antagonism is illustrated under the plant fitness equilibrium, in which the two extremes lead to either hybrid necrosis or heterosis. Practical proposition from the equilibrium model is that breeding efforts for combining enhanced disease resistance and high yield shall be achieved by balancing the two forces. Reverse breeding toward utilizing genomic data centered on immune components is proposed as a strategy to generate elite hybrids with balanced immunity and growth.

## Introduction

Hybridization between individuals of the same or different plant species leads to the formation of hybrids that contain a copy of each parental genome. This heterozygosity results in novel genetic combinations that were never found in the respective parental lines, and thus distinct phenotypes can be sometimes expressed in the hybrid (Mallet, 2007). Genetic interactions in the hybrid can result in beneficial phenotypes, of which the phenomenon is termed as heterosis or hybrid vigor (Shull, 1948; Schnable and Springer, 2013). On the contrary, such interactions can lead to detrimental phenotypes, as found in hybrid weakness or hybrid necrosis cases. Hybrid necrosis is a common phenomenon observed in plant hybrids featuring immune-related deleterious phenotypes (Bomblies and Weigel, 2007; Bomblies, 2009). The term reflects phenotypically recognizable damages in plant tissues with visible necrosis resulting from uncontrolled cell death and/or autoimmune-like symptoms including dwarfism, stunted growth, leaf crinkling, reduced fertility, and in severe instances death before transitioning to the reproductive phase (Bomblies and Weigel, 2007; Chae et al., 2014). The overall compromise in plant performances observed in hybrid necrosis cases is believed to reflect trade-offs between immunity and growth (Todesco et al., 2014; Chae et al., 2016; Świadek et al., 2017). Due to its drastic effect on survival and prevalence in F_1_ generations, hybrid necrosis is postulated to provide plant populations with a conducive mechanism to create post-zygotic barriers toward eventual speciation (Bomblies and Weigel, 2007).

### Hybrid Necrosis Under the Bateson-Dobzhansky-Muller Model and Negative Epistasis

**Figure 1:**
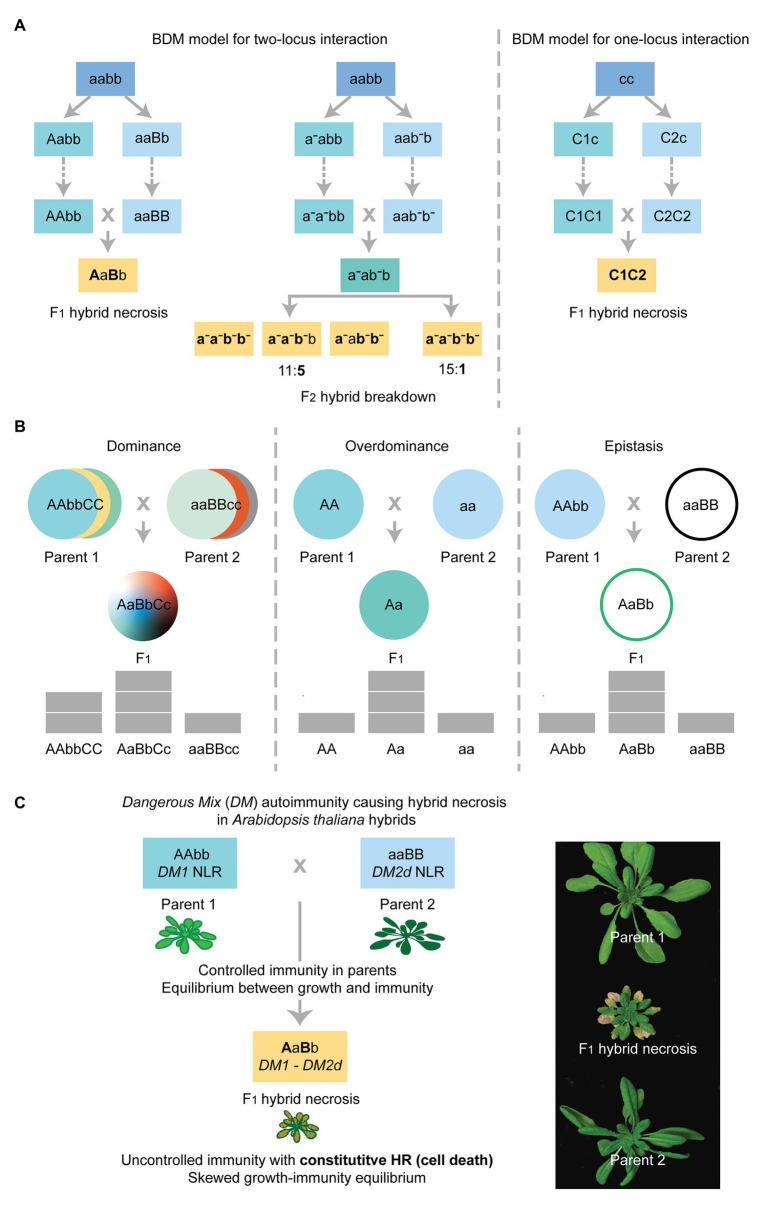
The genetic mechanisms of hybrid necrosis caused by negative epistasis explained by the Bateson-Dobzhansky-Muller (BDM) model. **(A)** The BDM model for two-locus or one-locus interaction triggering F_1_ hybrid necrosis and F_2_ hybrid breakdown in hybrid necrosis cases (yellow text-boxes). Ancestral status of alleles at indicated loci is uncapitalized and derived allelic status is indicated as capitalized for hybrid necrosis cases. Boldface letters indicate alleles that are under deleterious epistasis for hybrid necrosis and hybrid breakdown cases. The dotted arrows in each scheme indicate that numerous generations are required before the different allelic combinations represented in the colored boxes are fixed in independent lineages. In the cases of hybrid necrosis locus/loci, derived alleles behave as dominant or semi-dominant (Bomblies et al., 2007; Alcázar et al., 2009; Chae et al., 2014). In F_2_ hybrid breakdown, ancestral status and derived allelic status differ from each other by the presence of the upper dash for derived recessive alleles. Note that classical F_2_ hybrid breakdown cases manifest when the homozygous recessive state at two loci is met with the derived alleles (a-a-;b-b-) or the homozygous recessive state of one locus is combined with the heterozygous status of the other locus (a-a-;b-b or aa-;b-b-) in 15:1 or 11:5 segregation (Oka, 1957; Fukuoka et al., 1998; Okuno, 1999; Matsubara et al., 2007, 2014; Ichitani et al., 2012). **(B)** Genetic mechanisms of dominance, overdominance, and epistasis leading to phenotypes that respond to both qualitative (color) or quantitative (pyramid) traits. The first two mechanisms attribute the observed phenotype in the hybrid to additive effects, arising from allelic interactions between dominant over recessive alleles at various loci (dominance) or at a single locus (overdominance). Epistasis attributes any phenotype that deviates from the expected additive effect of two alleles to non-allelic interactions (i.e., two different loci). Note that the BDM model for two-locus interaction shown in **(A)** corresponds to epistasis, while overdominance explains the BDM model for one-locus interaction shown in **(A)**. **(C)** Left: Autoimmunity triggered by the *DM1/DM2d* hybrid necrosis pair. The two loci, *DM1* and *DM2d*, are innocuous in their respective parental lineages. The combination of *DM1* and *DM2d* in the *Arabidopsis thaliana* hybrid triggers a constitutive activation of defense mechanisms in the absence of pathogen attack, which results in the hypersensitive response (HR) and hybrid necrosis (Bomblies et al., 2007; Tran et al., 2017). Right: F_1_ hybrid displaying cell death in somatic tissues and the comparison to the respective parents.

Hybrid necrosis arises from novel genetic interactions in heterozygous backgrounds. This phenomenon often manifests in the F_1_ generation, but cases in the F_2_ have also been described. Numerous examples of hybrid necrosis in plants demonstrate that the dynamics of genetic incompatibilities found in F_1_ and F_2_ hybrids conform to the simple Bateson-Dobzhansky-Muller (BDM) model for two-locus interaction that had been formulated to explain the speciation mechanism (Figure 1A; Orr, 1996). The BDM model posits that independently evolved genetic variants are harmless in their respective parental lineages but the combination of a pair becomes incompatible in the hybrid (Figure 1A). Since inviable, inferior or infertile offspring are the outcome of such incompatibilities, such genetic mechanisms are considered to establish major postzygotic reproductive barriers between different lineages, hence promoting speciation (Orr, 1996).

A wider spectrum of phenotypes are found in hybrids as compared to the parents, including heterosis and hybrid necrosis, and the underlying causes can be generally explained by genetic interactions following the three schemes, namely (i) dominance, (ii) overdominance, and (iii) epistasis (Figure 1B). These three mechanisms can operate simultaneously, but independently, to allow the expression of different traits (Li et al., 2008; Huang et al., 2016). The dominance hypothesis is often favored to explain heterotic phenotypes, which attributes the expression of heterosis to the heterozygous allelic status at multiple loci in hybrids. The premise is that in parental lines phenotypic expression for a given trait is constrained by the sum of deleterious, recessive alleles. In heterotic hybrids, the introduction of beneficial or dominant alleles from both parents at these multiple loci breaks the recessiveness that had caused inferiority in the respective parental backgrounds, thus improving hybrid performance (Figure 1B, left; Hochholdinger and Hoecker, 2007). The overdominance hypothesis, on the other hand, attributes superior- or under-performance in the hybrid to the heterozygosity at a single locus as compared to the homozygous status in parents (Figure 1B, middle; Hochholdinger and Hoecker, 2007). Lastly, epistasis refers to non-additive genetic interactions deviating from the summation of allelic effects at the involved loci (Figure 1B, right; Fisher, 1919). It is necessary to point out, however, that other mechanisms such as epigenetics can control the expression of traits in hybrids (Fujimoto et al., 2018). For instance, experiments in *Arabidopsis thaliana* using epigenetic recombinant inbred lines (Epi-RILs) have shown that epialleles are inheritable and can explain phenotypic variation in certain developmental traits (Cortijo et al., 2014; Zhang et al., 2018). The BDM model for hybrid necrosis is certainly a well spelled-out case of negative epistasis involving two derived alleles from the involved genetic loci. In most hybrid necrosis cases, the derived alleles act as dominant or semi-dominant, in a dosage-dependent manner. There are hybrid necrosis cases only visible in the F_2_ generation, of which genetic scheme can be similar to the classical F_2_ hybrid breakdown, albeit with strong dose-dependent effects (Figure 1A). Genetic studies on hybrid necrosis cases have demonstrated that a pairwise epistatic relationship between alleles at two distinct loci inherited from each of the parents does condition autoimmunity in both F_1_ and F_2_ hybrid generations (Figure 1A, left; Bomblies et al., 2007; Alcázar et al., 2009; Chae et al., 2014). In addition, cases of hybrid necrosis involving different alleles at a single locus have also been identified. Such negative epistasis can be easily explained by a simple modification of the BDM model with a replacement of one unlinked locus with the different alleles of the same locus (Figure 1A, right; Alcázar et al., 2014; Chae et al., 2014). To conclude, the BDM model explains the majority of F_1_ or F_2_ hybrid necrosis cases as a result of negative epistasis. Due to the deleterious effect on hybrid performance, hybrid necrosis can serve as a conducive mechanism to create a reproductive barrier between two independently evolving parental lineages (Figure 1A).

### Autoimmunity as an Underlying Mechanism for Hybrid Necrosis

Numerous hybrid necrosis cases have been identified in crops of economic relevance such as wheat (Hermsen, 1963), interspecific hybrids of *Triticum-Aegilops* (Mizuno et al., 2010), rye (Ren and Lelley, 1988), lettuce (Jeuken et al., 2009), rice (Yamamoto et al., 2010), cotton (Phillips, 1977; Deng et al., 2019), and in natural germplasms of *A. thaliana* and *Capsella* species (Bomblies et al., 2007; Chae et al., 2014; Sicard et al., 2015). Because hybrid necrosis has been observed in a broad range of species (see review Bomblies et al., 2007), it is of major interest to define the causal genetic components and investigate if there is a common underlying mechanism. Accumulating fine-mapping data obtained from various hybrid necrosis cases in different species point out that this phenomenon arises from the uncontrolled activation of immune responses in the absence of pathogens. Such links to autoimmunity had already been suggested by Kostoff in 1930, who indicated that hybrid necrosis phenotypes resemble those triggered by pathogens and therefore suggested autoimmunity as a major cause of hybrid necrosis (Kostoff, 1930). However, it has only been over a decade since the causality was identified to the R protein families by molecular cloning (Krüger et al., 2002; Rooney et al., 2005; Bomblies et al., 2007; Alcázar et al., 2009; Chae et al., 2014). Nucleotide-binding and leucine-rich repeat (NLR) immune receptors in the majority and in some cases receptor-like proteins (RLPs) or receptor-like kinases (RLKs) families contribute to trigger autoimmunity in hybrids. NLRs constitute the major class of resistance genes (hereafter *R* genes) characterized to date, and plant genomes often carry hundreds of *R* gene homologs, some of which are highly variable even within a species (Sarris et al., 2016; Kourelis and van der Hoorn, 2018; Van de Weyer et al., 2019). It is thought that such variability reflects the need for the gene-for-gene hypothesis, in which a specific R protein recognizes a matching avirulent effector that can be highly variable in pathogen populations (Flor, 1971; Chen and Ronald, 2011). Because pathogens evolve rapidly, plant NLRs in a given population are under the pressure of diversifying to provide sufficient recognition-specificity for effectors, of which coevolution is best explained under the concept of an arms race between host and parasites (Holub, 2001; Borrelli et al., 2018). Such diversity, however, can result in a collateral conflict involving NLRs; when divergently evolved NLRs are found in combination with other immune components, one can miss-recognize the other and trigger autoimmunity (Chae et al., 2014).

In the past years, thanks to fine-scale genetics and well-annotated genomes, it has been made possible to identify hybrid necrosis triggering epistatic loci down to individual genes. The first causal link between hybrid necrosis and autoimmunity has been established in *A. thaliana* with the epistatic interaction between *DANGEROUS MIX1* (*DM1*) and *DANGEROUS MIX2* (*DM2*; Bomblies et al., 2007). Both *DM* genes are located in the polymorphic, multi-gene clusters encoding multiple NLRs, with *DM2* homologs having known isolate-specific resistance function to the adapted pathogen *Hyaloperonospora arabidopsidis* (*Hpa*; Figure 1C). Later, a systematic study carried out with over 6000 F_1_ hybrids generated from 80 *A. thaliana* parental accessions corroborated the notion that hybrid necrosis is commonly mediated by negative epistasis between highly polymorphic components of the immune system, such as NLRs (Chae et al., 2014). This large-scale investigation provides a species- and genome-wide insight on which immune components are more prone to generate predisposed alleles for autoimmunity. Interestingly, not all NLR loci turned out to contribute to the generation of autoimmunity-risk alleles but there exist particular genome loci recurrently spawning the risk alleles (Chae et al., 2014). Among such hybrid necrosis hot spots in the *A. thaliana* genome, the *DM2* cluster is the most prominent one producing multiple hybrid necrosis alleles interacting with a broad range of loci, including other NLR-containing clusters and the loci encoding enzymes (Alcázar et al., 2010; Chae et al., 2014; Tran et al., 2017). For instance, hybrids between L*er* and Kas-2 accessions of *A. thaliana* display mild-F_1_ hybrid necrosis with obvious phenotypes found in F_2_ and later generations, and the causal BDM pair is an NLR encoded by the *DM2* locus from L*er* and the SRF3 RLK from Kas-2 (Alcázar et al., 2009, 2010). There also seems to be a loyal partnership between BDM epistatic loci: multiple allelic pairs of *DM6* and *DM7* have been discovered to cause distinct classes of hybrid necrosis (Chae et al., 2014; Barragan et al., 2019). It has also been revealed that deleterious interactions can occur between different alleles at a single locus, such as *DM8* and *DM9*, as illustrated in Figure 1A (right; Chae et al., 2014). Recent analyses on the complex NLR encoding loci, of which dataset had been much improved by long-read sequences compared to Illumina short read-mapping based NLR assembly, revealed that the *DM* loci are definitely characterized with a high propensity of genomic rearrangements, including asymmetric cluster expansion and contraction in the *A. thaliana* natural accessions (Van de Weyer et al., 2019; Jiao and Schneeberger, 2020; Lee and Chae, 2020). Thus, a high correlation between the *DM* locus predisposition and structural variation involving copy number variations (CNVs) emerges as an obvious feature. Given that the causality often maps to a single gene in the *DM* multi-gene clusters, it is likely that the cluster expansion and contraction events promote to generate such deleterious alleles. Importantly, the large-scale hybrid necrosis survey in *A. thaliana* has not only provided the incidence rate of hybrid necrosis at about 2% but also established high-throughput platforms for identifying causal allelic variants under epistatic interaction. Once an allele frequency contributing to the same type of hybrid necrosis is determined under a large-scale crossing scheme, such as diallelic crosses, genome-wide association studies (GWAS) can be utilized to identify single-nucleotide polymorphisms (SNPs) tagging the causal variants (Barragan et al., 2019, 2020). It has been also found that geographic distances between two parental lines do not necessarily correlate with the chances of running into immune system incompatibilities, as was demonstrated by *DM1* and *DM2d* carriers, Uk-3/Uk-6 and Uk-1 coming from a small town in Germany, Umkirch (Mouchel et al., 2004; Bomblies et al., 2007).

In crops, a systematic study focused on autoimmune hybrids has not been carried out obviously due to its seemingly low economic and agricultural potential, but there are certainly a massive number of examples reported from breeding panels. In an interspecific hybrid of lettuce, a deleterious epistatic interaction involving *RPM1 INTERACTING PROTEIN4* (*RIN4*) and potentially an *R* gene has been identified to lead to a dosage-dependent hybrid necrosis (Jeuken et al., 2009). Later, the causal interacting *R* locus, *DM39*, was mapped to an NLR gene cluster (Christopoulou et al., 2015; Parra et al., 2016). The protein RIN4 is one of the most well studied immune components (guardee, see section Diversity of NLRs in Sequence and Function for details) in plants, of which integrity is being monitored by multiple *R* proteins. Importantly, the most extreme case of hybrid necrosis has been reported in cotton in which an NLR gene, *Le4* leads to F_1_ interspecific hybrid lethality in the cross between *Gossypium barbadense* and *Gossypium hirsutum* (Deng et al., 2019). The relevance of these findings in crops lies in the fact that a functional correlation could be made between hybrid necrosis alleles and disease resistance traits in the field. One of the most important unanswered questions is whether hybrid necrosis alleles can arise through the course of enhancing disease resistance either from selective breeding or through evolution in the wild. This connection is to be best addressed when hybrid necrosis research is carried out in crops with well-defined pathosystems and available breeding history such as in rice.

In this review article, we overview recent progress in hybrid necrosis research focusing on genetic and molecular details. Although initial works were favorably discussed under speciation mechanism, current research outcomes are heavily detailed in inbreeding species for obvious practicality in the pursuit of identifying causal genes. With a strong link to the plant immune system, we take an initiative to extend our view of hybrid necrosis to study plant performance in hybrids under the equilibrium of growth and immunity. We also attempt to revisit the cases of rice genetic incompatibilities responsible for various deleterious phenotypes found in F_1_ and F_2_ hybrids to examine if any of the reported criteria falls under the definition of immune system incompatibility. We propose that hybrid necrosis as a result of immune system incompatibility illustrates different levels of balancing act between immunity and growth. Thus, the characterization of hybrid necrosis in crops can guide breeding efforts to equilibrate resistance and yield to produce elite hybrids. This strategy will be highly informative in crops relying on hybridization as a major breeding method such as in rice. To this end, we explain in detail the molecular mechanism governing the autoimmunity in hybrids and suggest a way to mitigate autoimmunity to simultaneously enhance growth and resistance in breeding lines. Finally, we present the possible application of reverse breeding, a breeding technique that can rapidly deliver different types of mapping populations useful for the study of hybrid necrosis and heterosis.

## Hybrid Necrosis and Heterosis: A Matter of Extremes

### Trade-off Between Immunity and Growth

The trade-off between immunity and growth in plants is thought to occur as a result of prioritizing resource allocation to either of the two processes. An optimized equilibrium in response to external or internal factors would be achieved to ensure plant fitness and health (Huot et al., 2014). Optimal immune responses certainly promote the survival of plants in the time of needs, such as to fend off pathogen attack. For instance, under a certain avirulent bacterial pathogen load, it has been demonstrated that *A. thaliana* plants carrying the matching NLR will initialize an acute immune response, also known as effector-triggered immunity (ETI). Despite the fact that such a response disposes infection sites *via* activating local cell death, known as the hypersensitive response (HR), these plants display overall enhanced fitness as compared to those that do not carry the corresponding NLR. Indeed, regarding lifetime fitness, resistant lines can significantly outperform susceptible individuals when both are infected with pathogens. When total silique length was used as a proxy for lifetime fitness, it has been demonstrated that resistant *A. thaliana* isolines carrying an allele of *RPS5* (RPS5+) NLR that confers resistance to the avirulent *Pseudomonas syringae* strain show enhanced values by 9.6–32% in overall fitness measurement compared to susceptible lines (RPS5−) under the semi-controlled glasshouse condition (Gao et al., 2009). These findings support the idea that robust resistance conferred by an NLR for a matching pathogen enhances fitness if plants are under pathogenic attack.

The fact that energetic resources will be consumed once ETI is activated can logically explain a growth penalty. The complexity of this trade-off, however, largely resides in the fact that a fitness cost is associated with resistance even in the absence of obvious pathogen load. The pioneering work from Bergelson’s group provided strong quantitative evidence for this (Tian et al., 2003). The *RPM1* NLR is an *R* gene in *A. thaliana* that confers resistance against the *P. syringae* strain DC3000 carrying matching avirulence (Avr) effectors (Dangl et al., 1992; Innes et al., 1993; Bisgrove et al., 1994). To examine the cost that *RPM1* resistance would exact, susceptible host lines were transformed with the *RPM1* resistant allele to create a total of four isogenic lines (*RPM1*+). When *RPM1*+ lines were compared against susceptible lines (*RPM1*−) in a field trial where the avirulent strain was absent, *RPM1*+ showed lower shoot biomass and an average of 9% seed-set reduction as compared to *RPM1*−. Interestingly, resistant and susceptible alleles of *RPM1* coexist in the global populations of *A. thaliana*, indicating that balancing selection acts on to maintain both alleles. The signature of balancing selection endorses the premise that *RPM1* confers both a fitness advantage and a fitness cost depending on the presence and absence of the matching pathogens (Tian et al., 2003). A similar experimental setup has shown that carriers of an NLR, *RPS5* (*RPS5*+), that confers resistance to *P. syringae* carrying *AvrPphB* or its homologs, suffer from a yield penalty in comparison to lines carrying the susceptibility allele (*RPS5*−; Karasov et al., 2014). Field trials performed with the *RPS5*+ and *RPS5*− that were not exposed to *P. syringae* showed a reduction in the seed-set between 5 and 10.2% in the *RPS5*+ lines in comparison to *RPS5*− lines. Supporting the fitness cost associated with *RPS5* resistance, a balanced polymorphism for *RPS5* has also been observed (Tian et al., 2002). In both cases, fitness costs associated with the NLRs led to about 9–10% decrease in seed production in the absence of obvious pathogen infection, and such costs are not likely to be explained merely by the metabolic cost of RPM1 and RPS5 synthesis. There can be alternative reasons for the observed fitness costs in both experimental setups. First, although the particular pathogen that is thought to trigger the HR was not found during the field trials, it is possible that other, yet unknown, effectors from several pathogen species can interact with RPS5 and RPM1, triggering a constitutive defense response that would negatively impact growth (Karasov et al., 2014). RPM1 in particular has been shown to be a common target of multiple effectors in laboratory conditions (Grant et al., 1995; Kapos et al., 2019; Ray et al., 2019). Such diffused *R-Avr* interactions, as opposed to Flor’s gene-for-gene hypothesis, would also explain relatively high frequencies of RPM1+ and RPS5+ natural accessions of *A. thaliana* despite the cost (Stahl et al., 1999; Rose et al., 2012; Karasov et al., 2014). Second, RPM1 and RPS5 may negatively interact with other *R* genes in the introduced background (Tian et al., 2003). Such type of interaction could very well correspond to negative epistasis following the BDM model, but further research is required to confirm whether there are partners for *RPM1* or *RPS5* exerting mild autoimmunity. Intriguingly, *RIN4*, an important host partner of *RPM1*, has been identified as a hybrid necrosis gene in lettuce interacting potentially with an NLR gene, indicating the BDM epistasis may occur through a host protein that such costly NLRs surveil. Overall, these findings imply that the composition of *R* gene repertoire in a given population determines the cost exerted to affect overall fitness because a fraction of costly *R* genes would bring down yield even under seemingly non-pathogenic conditions. In addition, these results highlight the importance of controlling such costly *R* genes in the host germplasm.

Since a well-managed trade-off can reward breeding efforts, there have been substantial research endeavors to uncover regulatory mechanisms that coordinate growth and immune responses (Karasov et al., 2017). Immune responses supposedly divert substantial amounts of resources that otherwise would be invested in growth, and multiple hormonal pathways have been under investigation to establish the link between growth and immunity (Purrington, 2000; Huot et al., 2014). Several studies have pointed out that crosstalks between different hormonal pathways are instrumental in shifting the balance between growth and defense (Glazebrook, 2005). Generally, but not exclusively, it is considered that growth-promoting hormones, including cytokinins, brassinosteroids (BR), auxins and gibberellins (GA), act against defense-promoting hormones such as ethylene (ET), salicylic acid (SA), and jasmonates (JA; Liu et al., 2008; Tsuda et al., 2009; Clouse, 2011; Emenecker and Strader, 2020). However, direct molecular links between an individual or a suit of hormonal pathways and immune responses still remain to be investigated in-depth (An and Mou, 2011; Chen and Ronald, 2011; De Vleesschauwer et al., 2014; Lozano-Durán and Zipfel, 2015). A mechanistic insight about the trade-off involving NLR-mediated immunity has been provided by the work in rice, demonstrating functional dependency between the fungal resistance NLR *Pik-H4* and its interacting protein OsBIHD1 that encodes a homeodomain transcription factor (Liu et al., 2017). Physical interaction between Pik-H4 and OsBIHD1 is necessary for ETI, supporting the importance of OsBIHD1-mediated control in immunity. During infection, OsBIHD1 launches a growth-inhibiting process by activating reactions that catabolize the growth-promoting BR. OsBIHD1, in addition, facilitates ET synthesis, which in turn enhances the NLR-mediated immunity. Interestingly, not only the overexpression but also the knockout of *OsBIHD1* leads to dwarfism, suggesting that OsBIHD1 is a critical factor coordinating growth potential through the regulation of other growth-promoting hormones or processes (Liu et al., 2017). This work elegantly describes how a single factor is able to modulate ET-BR crosstalk in response to NLR-mediated defense and how such modulation affects plant growth. Engagement of such an important growth regulator into the NLR action during infection appears to be a way to effectively reallocate resources.

On the other hand, untimely or uncontrolled activation of immunity often results in a decrease in plant fitness. Indeed, numerous lesion-mimic mutants identified in *A. thaliana* and rice illustrate a skewed balance toward constitutive activation of immune responses, resulting in growth compromise typically expressed with dwarfism, leaf curling, stunted roots, and death (Heil and Baldwin, 2002; Zhu et al., 2020). Some of these lesion-mimic mutants in *A. thaliana* were further characterized to reveal the contribution of NLR to such autoimmunity (van Wersch et al., 2016). For instance, a gain-of-function mutation in one of the *DM1* homolog is named *SSI4* (*SUPPRESSOR OF SALICYLIC ACID INSENSITIVITY OF NPR1-5, 4*) characterized with obvious autoimmune symptoms (Shirano et al., 2002). The most well-characterized autoimmune mutant would be *snc1* (*suppressor of npr1-1, constitutive 1*) in *A. thaliana*, which had been identified with its dominant allele suppressing defects of *npr1-1* plants in SA pathway regulation (Li et al., 2001; Zhang et al., 2003). Both dominant mutations fall in the domain of the two NLRs important for regulating its activity. Another lesion mimic mutant in *A. thaliana*, *acd11* clearly shows autoimmunity-related compromise in growth due to a mutation in the gene involved in sphingolipid modification (Brodersen et al., 2002). However, later it has been demonstrated that the cell-death observed in *acd11* can be suppressed by mutations in *LAZ2*, a gene that encodes a histone-methyltransferase, which in turn controls the expression of an NLR named *LAZ5* (Palma et al., 2010). Likewise, *ACD6*-mediated growth compromise commonly observed in naturally occurring autoimmune accessions has recently been shown to require particular natural alleles of *SNC1* (Todesco et al., 2010; Zhu et al., 2018). Similar contributions of *SNC1* and other NLR alleles had been observed to regulate the class of *BONZAI* mutants characterized by dwarf phenotypes (Hua et al., 2001; Yang and Hua, 2004; Li et al., 2009). The involvement of NLRs in these lesion-mimic mutants strongly suggest that cell death execution in these mutants likely is mediated by NLRs, and such mutants indeed reveal regulatory mechanisms tightly controlling NLR activity to balance the equilibrium with growth promotion.

The above-described findings hint us to assess NLR function in lesion-mimic mutants in other species. For instance, a rice lesion-mimic mutant collection includes *Spl26* and *spl17* exhibiting chlorosis and cell death, as well as conferring resistance to both fungal blast and bacterial blight (Wu et al., 2008). The penalty on plant fitness is high in these mutants such that homozygosity of the dominant lesion-mimic mutations prevents them from reaching maturity and that the trans-heterozygosity between them makes plants lethal (Wu et al., 2008). Due to the enhanced resistance conferred by these rice mutants, one can easily hypothesize a link to NLR-mediated strong immune responses as the cause for the severe autoimmunity in rice. It would be a rewarding research agenda to further investigate the lesion-mimic mutants in rice with beneficial field resistance traits, which will allow us to examine how such enhanced resistance is achieved and examine the relative importance of causal factors in the plant immune system. In rice, constitutive activation of defense signaling and growth inhibition can also be simultaneously mediated by WRKY transcription factors. Overexpressing lines of *OsWRKY31* show constitutive activation of defense-related genes and enhanced resistance to rice blast, however, growth penalty is observed in lateral root formation and elongation (Zhang et al., 2008). The growth-deficient phenotype has been attempted for rescue by the administration of exogenous auxin, but due to a reduced auxin sensitivity in this line the trial failed. The result suggests that OsWRKY31 could act in the signal transduction of both the auxin and defense responses (Zhang et al., 2008), offering promising grounds to investigate the role OsWRKY31 in growth and immunity. WRKY transcription factors are known for its general function in defense in plants such as OsWRKY03 acting upstream of NPR1 (Liu et al., 2005), WRKY70 in *A. thaliana* mediating crosstalk between JA and SA (Li et al., 2004), and most importantly, decoy WRKY domains integrated to NLRs (Cesari et al., 2014a; see NLR-IDs in section Mechanisms of NLR-Mediated Autoimmunity Responsible for Hybrid Necrosis for detail). An additional functional link between WRKY and NLRs in rice has been established with five rice NLR proteins (Piz-t, Pib, Pi36, Pit, and Pita) interacting with WRKY45 and WRKY66 (Liu et al., 2016). Thus, it seems that growth-affecting defense pathways known in rice shall be revisited to further refine their molecular details to reveal regulatory mechanisms for the trade-off between growth and immunity.

### Yield Penalty Accompanying Disease Resistance Traits Reflects the Trade-off

In breeding panels, the above-mentioned trade-off has been conceptualized under yield penalties associated with disease resistance traits. Low yield, due to reduced grain weight and/or a general reduction in plant growth, reflects a skewed balance favoring the maintenance of resistance traits that would protect crops from pathogenic infection at the expense of yield (Ning et al., 2017). Rice yield has steadily increased since the early 1960s in Asian countries like China, Vietnam, Indonesia, and India (Qian et al., 2016). The success of hybridization programs and the identification of major quantitative trait loci (QTLs) for heterosis along with their systematic incorporation to the existing elite lines are certainly responsible for major increases in rice production (Qian et al., 2016). Despite breeding success in yield traits *per se*, sustaining high yield in the deployed lines is evidently facing problems due to pest management. Indeed, rice production is being seriously affected by several pathogens such as the insect brown planthopper (*Nilaparvata lugens*), rice blast fungus (*Magnaporthe oryzae*), rice bacterial leaf blight (*Xanthomonas oryzae* pv. *oryzae*), rice bacterial leaf strike (*X. oryzae* pv. *oryzicola*), and Tungro virus (Azzam and Chancellor, 2002; Bottrell et al., 2012; Liu et al., 2014). Climate change is predicted to escalate yield losses as both disease resistance and yield traits are optimized for certain environmental conditions (Qian et al., 2016; Muehe et al., 2019). Thus, engineering robust and durable disease resistance traits with a minimized yield penalty in elite rice hybrids is an urgent matter.

Unfortunately, the catalog of field-effective *R* genes in rice rather remains short-listed. Although hundreds of QTLs for resistance against different pathogens across taxa have been identified in experimental populations, only a handful of *R* genes have been used for hybrid breeding (Liu et al., 2014; Qian et al., 2016; Li et al., 2019). For instance, among ~30 genes cloned to date, most of which encoding NLRs (Wang et al., 2017), only a limited number of loci, for example, the locus encoding *Pi9/Pi2/PigmR*, have been under frequent use due to their ability to confer durable and/or broad-spectrum resistance for rice blast isolates (Cesari and Kroj, 2017; Deng et al., 2017; Tian et al., 2019). Although recent reports have successfully identified over a hundred potential new *R* genes against different isolates of rice blast out of hundreds of cloned rice NLRs present in Tetep and other blast-resistant cultivars (Zhang et al., 2015; Wang et al., 2019c), introgression of the newly discovered in elite lines have not yet been reported. Given that *Xa2*, a favorable blight *R* gene with no obvious yield penalty is of Tetep origin (Ogawa et al., 1991), genome-assisted *R* gene discovery from a cultivar with broad-spectrum resistance offers a promising path. In addition, *Xa4* and *Xa21*, along with *Xa2*, are non-NLR R proteins and characterized by their durable nature and least yield penalty (Ogawa et al., 1991; He et al., 2006; Kottapalli et al., 2010; Ning et al., 2017), suggesting potential routes to broaden our search for functional R proteins other than NLRs. On the other hand, achieving durable resistance for pathogens like rice blast is still challenging as resistance conferred by single *R* genes would often break down within 3–5 years due to rapid cycling of pathogens that outpace the host immune system adaptation (Devi et al., 2015). To broaden the spectrum and durability of blast resistance, several *R* genes have been combined in the same background (Wang et al., 2019c). Although the current endeavor focuses on pyramiding *R* genes to promote both broad-spectrum and durable resistance in commercial lines (Hu et al., 2012; Miah et al., 2013; Xiao et al., 2017; Sabar et al., 2019), pyramiding resistance genes for any of the major rice pathogens in a novel genetic background is expected to encounter negative epistasis between newly assembled *R* gene combinations to a certain extent (Figure 1C). In addition, usually each set of *R* genes has been cloned based on the specific resistance to a single pathogen species or even to an isolate (Flor, 1971). Furthermore, it is technically tedious and costly to incorporate several *R* genes in new breeding lines every few years (Li et al., 2019). The introgression of *R* genes from donor lines does not always result in enhanced resistance in the recipient background due to an interference in immunity from the existing immune components (Hurni et al., 2014; Stirnweis et al., 2014; Kim et al., 2019). Finally, only several *R* genes are known not to affect yield after introgression in the commercial lines (Ning and Wang, 2018).

Negative effects on yield born by *R* gene pyramiding in crops are well documented (Ning and Wang, 2018). For this reason, introgression of new *R* genes in rice elite varieties is very often accompanied by a thorough evaluation of possible yield penalties (Xiao et al., 2017). Due to the correlation observed between *R* gene pyramiding with a decrease in yield, it is tempting to speculate that rice elite varieties that produce high-yielding hybrids have not only accumulated heterotic traits but also dispensed a suit of costly or unnecessary *R* genes during the breeding. Therefore, it is plausible that these lines have been selected for *R* gene loci that had accumulated mutations abrogating or compromising the resistance function. Supporting this proposal, an impressive large-scale GWAS on rice heterosis revealed that breeding strategies for increasing yield inadvertently selected for susceptibility alleles for blast and leaf-blight resistance genes in rice. Huang et al. (2015) analyzed 1,495 elite hybrid and their respective inbred parental lines of *Oryza sativa* spp. *indica* and *O. sativa* ssp. *japonica* to investigate the genetic architecture governing heterosis in rice hybrids mainly bred in 1990s and 2000s. This study has identified major loci contributing to heterosis with large effects on certain traits, including disease resistance and grain quality together with multiple small-effect loci for grain yield, thus presenting a comprehensive view on trait fixation during domestication. One of the most striking results from this work regarding the link between immunity and growth is that high yield in hybrids is associated with the accumulation of susceptibility alleles. In the evaluated hybrids, the frequency of the resistant alleles for the locus *Pi2/Pi9* for rice blast and a locus associated with resistance to leaf-blight in chromosome 6 was 3.7 and 2.6%, respectively, which implies that the accumulation of susceptibility alleles had resulted in positive effects on yield production (Huang et al., 2015).

Accumulating susceptibility alleles on known *R* genes for rice blast and leaf-blight during breeding suggests that there had been a selection pressure favoring susceptibility alleles at those loci. Potentially, the absence or low number of avirulent strains for these resistances as well as fitness cost innate in such alleles might have rendered the resistant carrier generating low performing hybrids than the ones carrying a susceptible allele when used as parents. Unlike natural populations where such resistant and susceptible alleles are being maintained by balancing selection due to stochastic selective pressure imposed by pathogens (Tian et al., 2003; Karasov et al., 2014), selective breeding would favor seemingly higher-yielding ones at the expense of resistant alleles. These examples suggest that the trade-off between growth and immunity had played a critical role in directing the *R* gene repertoire in crops to be streamlined only for an immediate purpose. The artificial selection against resistant alleles of no immediate benefits would make the elite germplasms susceptible to diseases that can be easily resisted by their wild relatives. Less diversity in *R* genes in a population would increase the vulnerability to new pathogens, while the introduction of a new immune component such as an *R* gene becomes cumbersome. Problems in readapting *R* genes from wild relatives in the introgressed cultivated lines are evident, which caught not only breeders attention but also researchers who investigated complexity in the plant immune system. The first scientific report for a clashing immune component with the introgressed *R* gene appeared with the identification of Rcr3, an important immune papain-like cysteine endoprotease in tomato, that conflicted with the *Cf-2 R* gene from wild tomato *Lycopersicon pimpinellifolium* (Krüger et al., 2002). Negative epistasis clearly explains the incompatibility between *Rcr3* and *Cf-2*, triggering uncontrolled cell death and compromise in growth. The fine genetic analysis revealing epistasis in tomato made a foundation for hybrid necrosis studies to be established under the BDM model.

### Hybrid Necrosis Exemplifies a Trade-off Between Immunity and Growth

An obvious trend observed for hybrid necrosis cases in *A. thaliana* and other species is that uncontrolled immune responses negatively impacts on overall plant performance. The effects could range from mild compromise in growth to severe tissue necrosis and distortion in developmental programming (Alcázar et al., 2009; Jeuken et al., 2009; Yamamoto et al., 2010; Chae et al., 2014; Todesco et al., 2014; Sicard et al., 2015; Deng et al., 2019). In most hybrid necrosis cases, plants do not reach maturity to produce seeds. The characterization of overt hybrid necrosis in multiple plant species thus faces challenges in obtaining viable seeds to generate mapping populations unless advanced crossing schemes are engaged. Nonetheless, genetic architecture and molecular mechanisms underlying hybrid necrosis have been discovered since the detrimental autoimmune symptoms are temperature sensitive; changes in temperature can drastically suppress the symptoms and F_2_ progeny becomes available (Bomblies et al., 2007; Jeuken et al., 2009; Muralidharan et al., 2014). Our current understanding on the link between uncontrolled immunity and developmental defects and growth compromise still remains to be descriptive and further molecular characterization to define the connection is in need. Considering that the major mechanism underlying hybrid necrosis has been unequivocally attributed to autoimmunity only in recent years, it is very likely that the incidence of hybrid necrosis cases in rice and other crop species have been so far overlooked. The lettuce *RIN4* is an example of the discovery of epistasis upon revisiting the previous QTL mapping results for disease resistance traits under scrutiny (Jeuken et al., 2009). Therefore, to estimate the frequency of hybrid necrosis in crops, it would be necessary to reassess the data available from the literature and examine the cases of deleterious phenotypes observed in F_1_ and F_2_ hybrids to address how many of those could be contributed by immune genes and thus categorized under hybrid necrosis stemming from negative epistatic loci.

Deleterious phenotypes in rice hybrids have been reported as early as in 1957 by Oka, who described two different phenomena: hybrid inviability in the F_1_ generation and hybrid breakdown in F_2_. Oka established that dominant alleles cause plant death (Oka, 1957), which essentially conveys the concept of an allelic interaction following deleterious epistasis. In addition, he demonstrated that the genetic cause of the F_2_ hybrid breakdown was due to a set of duplicated genes in which the combination of recessive alleles leads to poor growth, proving a classic Michael Lynch’s prediction of hybrid incompatibilities arising from the combination of duplicate genes that had followed the independent fate (Lynch and Conery, 2000). Hybrid incompatibility due to negative epistasis between duplicated copies of the histidinol-phosphate aminotransferase gene which codes for HPA, a protein important in the biosynthesis pathway of histidine, has also been reported in *A. thaliana* (Bikard et al., 2009). The framework of hybrid weakness in rice prompted researchers in the following years to pursue more cases and characterize pairs of loci responsible for the range of hybrid weakness phenotypes (Table 1). Such research endeavors have identified some of the incompatibility loci to be mapped or linked to the *R* gene family (Table 1). For instance, *Hw4* was found to induce hybrid weakness when interacting with *Hw3*, which encodes for a putative calmodulin-binding protein (CaMBPs). We found it intriguing as CaMBPs are involved, among other processes, in plant defense mechanisms (Ali et al., 2003; Fu et al., 2013). Interestingly, further linkage-mapping analysis defined that *Hw3* was linked to *Pi1*, an allele of the *Pika* locus that confers resistance to blast, although it turned out that *Pi1* and *Hw3* was not the same gene (Hua et al., 2012; Fu et al., 2013). These findings show that *Hw3* and *Hw4* are both the causal genes of the hybrid weakness phenotype observed in rice, although the role played by components of the immune system in this genetic background and by the linkage between *Hw3* and the *R* gene is yet to be investigated. Moreover, the work of Yamamoto et al. (2007, 2010) has undoubtedly established the correlation between hybrid necrosis and autoimmunity in an *indica-japonica* F_2_ rice hybrid. Indeed, this particular genetic incompatibility case was investigated under the BDM-epistasis model, revealing that the interaction between two recessive genes, *hbd2* that codes for a casein kinase I (CKI1) and *hbd3* that is mapped to an NLR gene cluster region, is responsible for the reduced height and number of tillers in the autoimmune lines (Yamamoto et al., 2010). Interestingly, the deleterious allele effect of *hbd2* has been attributed to one amino acid change, indicating that a reproductive barrier between varieties of rice can be easily established due to autoimmunity (Yamamoto et al., 2010).

**Table 1:** Summary of hybrid weakness genetics in rice.

Hybrid	Phenotype	F	Genetics of the incompatibility	Causal gene	Reference
Intersubspecific *indica* × *japonica* hybrids	Sterility	F_1_	Intra-allelic and intra-genic interaction at the *S5* locus, which encodes 3 tightly linked genes.	*S5* encodes an Aspartic protease, potentially involved in disease resistance signaling and programmed cell death in reproductive tissues.	Yanagihara et al., 1995; Chen et al., 2008; Yang et al., 2012
Teqing × *O. rufipogon Griff*. hybrids	Autoimmunity. Interrupted root formation and impaired growth.	F_1_	Interaction *Hwi1* and *Hwi2*	*Hwi1* comprises 2 LRR-RLK genes, *25L1* and *25L2*. *Hwi2* encodes a putative subtilisin-like protease.	Chen et al., 2013, 2014
Intraspecific *indica* hybrids Taifeng A × V1134	Retarded growth, reduced panicle number, pale green leaves with chlorotic spots.	F_1_ F_2_	Two dominant loci: *Hw3* (From V113) and *Hw4* (from Taifeng)	*Hw3* encodes a putative calmodulin-binding protein.	Fu et al., 2013
Nipponbare × Jamaica hybrids	Compromised root growth (severe hybrid necrosis), difficulties maintaining root apical meristem (RAM), and shoot apical meristem (SAM). Rolled leaves and a short stature. Arrested leave development in embryos. Dwarfism, delayed flowering.	F_1_ F_2_	Interaction between *Hwc2* and *Hwc1*, with *Hwc1* being semi-dominant and having a stronger dosage effect than *Hwc2*.	*Hwc1* (possible candidates are orthologs to *LEUNIG* or *STYLOSA Arabidopsis thaliana* genes) Resistance genes linked to *Hwc2 locus: Pikahei-1(t), Pi39(t), Xa1, Xa2, Gm7*.	Ichitani et al., 2007; Saito et al., 2007; Kuboyama et al., 2009
Intersubspecific hybrids Sasanishiki (*japonica*) × Habataki (*indica*).	Reduction in the number of panicles per plant and in culm length	F_2_	Set of complementary recessive genes, *hbd2* and *hbd3*	N/A	Matsubara et al., 2007
Intersubspecific hybrids Koshihikari (*japonica*) × Habataki (*indica*)	Reduced growth, reduced number of tillers.	F_2_	Interaction between two recessive genes, *hbd2* and *hbd3*	*hbd2* (*deleterious CKI1*) and *hbd3* (mapped to NLR cluster region)	Yamamoto et al., 2007, 2010
Intraspecific *japonica* hybrids J-321 × J-147	Chlorosis and plant death	F_2_ F_3_	Two duplicated recessive loci: *HCA1* and *HCA2*	N/A	Sato and Morishima, 1988; Ichitani et al., 2012
Reciprocal hybrids of *Oryza perennis* (*barthii*) × related *Oryza* spp.	Embryo lethality, compromised growth of survivals.	F_1_	Dominant lethal alleles at two loci	N/A	Chu and Oka, 1970
Intraspecific *Indica* hybrids	Chlorosis, arrested root growth and development. Cell death, chlorosis, growth termination.	F_2_ F_1_	Recessive alleles at one locus or two loci. Interaction between weakness-causing alleles of *HWA-1* and *HWA-2*.	N/A	Oka, 1957; Ichitani et al., 2011; Shiragaki et al., 2019
Interspecific hybrids Koshihikari (*japonica*) × wild species *Oryza nivara*	Compromised growth and die before heading.	F_2_	Locus *hbd1(t)* from *O. nivara* interacting with other locus/loci in Koshikari background.	N/A	Miura et al., 2008
Intersubspecific hybrids Asominori (*indica*) × IR24 (*japonica*)	Sterility	F_2_	Epistasis between three unlinked loci *Hsa1*, *Hsa2*, *Hsa3*. *Hsa1 indica* locus containing *Hsa1a* and *Hsa1b* in homozygosity causes female sterility in *japonica*.	N/A	Kubo and Yoshimura, 2005; Kubo et al., 2016
Intersubspecific hybrids IR24 (*japonica*) × Asominori (*indica*)	Poor growth, complete sterility	F_2_	Duplicated recessive genes *Hwe1* and *Hwe2*. A rice blast resistance gene is located near *hwe1*.	N/A	Kubo and Yoshimura, 2002
Intraspecific hybrids Sanghaehyanghe olua (*japonica*) × Aranghyangchalb yeo (*japonica*)	Short, chlorotic plants with wilted leaves. Slow growth.	F_1_	Possible interaction between *Hwc1*, *Hwc2*, and *Hwc3*. *Hwc3* is cloned from Aranghyangchalbyeo. Sanghaehyangheolua carried *Hwc1*.	*Hwc3* encodes a LRR-containing protein with sequence similarity to *Xa1*, an NLR gene.	Yoshimura et al., 1998; Ichitani et al., 2007; Nadir et al., 2019
Intersubspecific *indica-japonica* hybrids “Tachisugata” and “Hokuriku 193”	Reduced growth	F_2_	Epistasis between two recessive alleles *hbd4*, carried by Tachisugata and *hbd5* carried by Hokuriku 193.	N/A	Matsubara et al., 2014
Intersubspecific *japonica-indica* reciprocal hybrids of Sasanishiki and other *japonica* cultivars and Col.no.15	Reduced growth and number of tillers	F_2_	*hwd1* (*Col.no.15 indica*) and *hwd2* (Sasanishiki-*japonica*) as double-recessive homozygous or homozygous for one locus and heterozygous for the second locus.	N/A	Okuno, 1985; Fukuoka et al., 1998; Okuno and Fukuoka, 1999

The number of underperforming hybrids described in the literature (Table 1) indicates that this phenomenon has an appreciable incidence in rice, which requires further attention for various reasons. First, we cannot exclude the possibility that hybrid necrosis has played a role in the observed underperformance. During rice domestication, disease resistance traits have been primarily selected within breeding panels of a region. This suggests that an imminent local pathogen load may have imposed a strong pressure selecting for a robust *R* gene in the bred lines. As has been demonstrated in hybrid necrosis cases, a new combination of *R* repertoire could result in underperforming hybrids, which may have not yet been rigorously classified under the hybrid necrosis category. As genetic architecture is simple, cases under BDM epistasis would easily generate lists of causal loci. In addition, it has been shown that in permissive or close-to-permissive temperature ranges the inferior hybrid phenotypes are alleviated (Saito et al., 2007; Fu et al., 2013; Chen et al., 2014; Muralidharan et al., 2014). Therefore, the characterization of underperforming hybrids under different environmental conditions and genetic identification of causality are relevant for improving hybrid performance in general. Finally, hybrid necrosis, not only severe but also mild ones, would preclude the expression of heterotic phenotypes. We believe that cryptic heterosis can be easily released if deleterious effects from a degree of autoimmunity are managed. We speculate that the negative correlation between immunity and plant performance, in particular growth and yield, is an important bottleneck for achieving heterosis in hybrid rice, which shall be extensively investigated with the underperforming hybrids. Not all underperformance would be attributed to the trade-off between growth and immunity, but addressing the proportional contribution made by elevated immunity to a decrease in growth can be effectively carried out using such rice hybrid germplasms. For this reason, we propose that the heterotic expression of agricultural traits can be improved when a degree of autoimmunity triggered by immune system conflicts is properly defined and mitigated.

### Fitness Equilibrium Model in Plants: Anti-hybrid Necrosis as Part of Heterosis?

The idea of mitigating autoimmunity in hybrids to increase overall fitness posits that the equilibrium between growth and immunity is an important factor determining plant performances. How such an equilibrium can be regulated and how the overall immunity of an individual limit the manifestation of heterotic traits remains to be determined. One might easily question if dampening residual or basal immunity level by systematically knocking down prominent immune receptor genes would indeed increase plant performances and yield under no obvious pathogenic loads. Such a systematic approach has not been embarked on; however, it appears that nature has embraced the needs to evolve certain microRNAs that can target multiple NLRs at a time to reduce NLR activity by and large (Zhai et al., 2011; Shivaprasad et al., 2012; Zhang et al., 2016). In addition, the expression of a cohort of NLRs clustered in a multigene cluster can be coregulated through the action of small-RNAs produced within the cluster (Yi and Richards, 2007). The complexity of such regulatory mechanisms as well as the genome studies focused on NLR evolution in different species at least provides us with the breadth and intricacy of NLR diversity present in natural populations (Van de Weyer et al., 2019; Lee and Chae, 2020; Seong et al., 2020). Although cataloging NLRs present in a species would not pinpoint which NLRs and how many of them are cost-exacting or predisposed to trigger autoimmunity in hybrids, detailed analysis of the pan-NLRome might inform us which of them had undergone either positive or negative selection and are associated with other traits than disease resistance, such as yield. Due to the poor-mapping quality of short reads, GWAS studies or other SNP-based genome-wide scans certainly have missed information covering complex NLR loci, often tandemly repeated with a massive structural variation. An improved assembly by whole genome sequencing with longer reads as well as the targeted assembly of NLR genes will greatly improve the resolution of trait mapping and genotype-phenotype association. In this regard, rice hybrids on the other hand offer a great system to investigate the potential contribution of hybrid incompatibilities of the plant immune system to limit the expression of heterosis. The exceptional amount of field-collected phenotype data in rice are not comparable to those existing in any other species, which will become a crucial component in the attempts to associate the phenome to immune-centered genotype platforms in rice.

**Figure 2:**
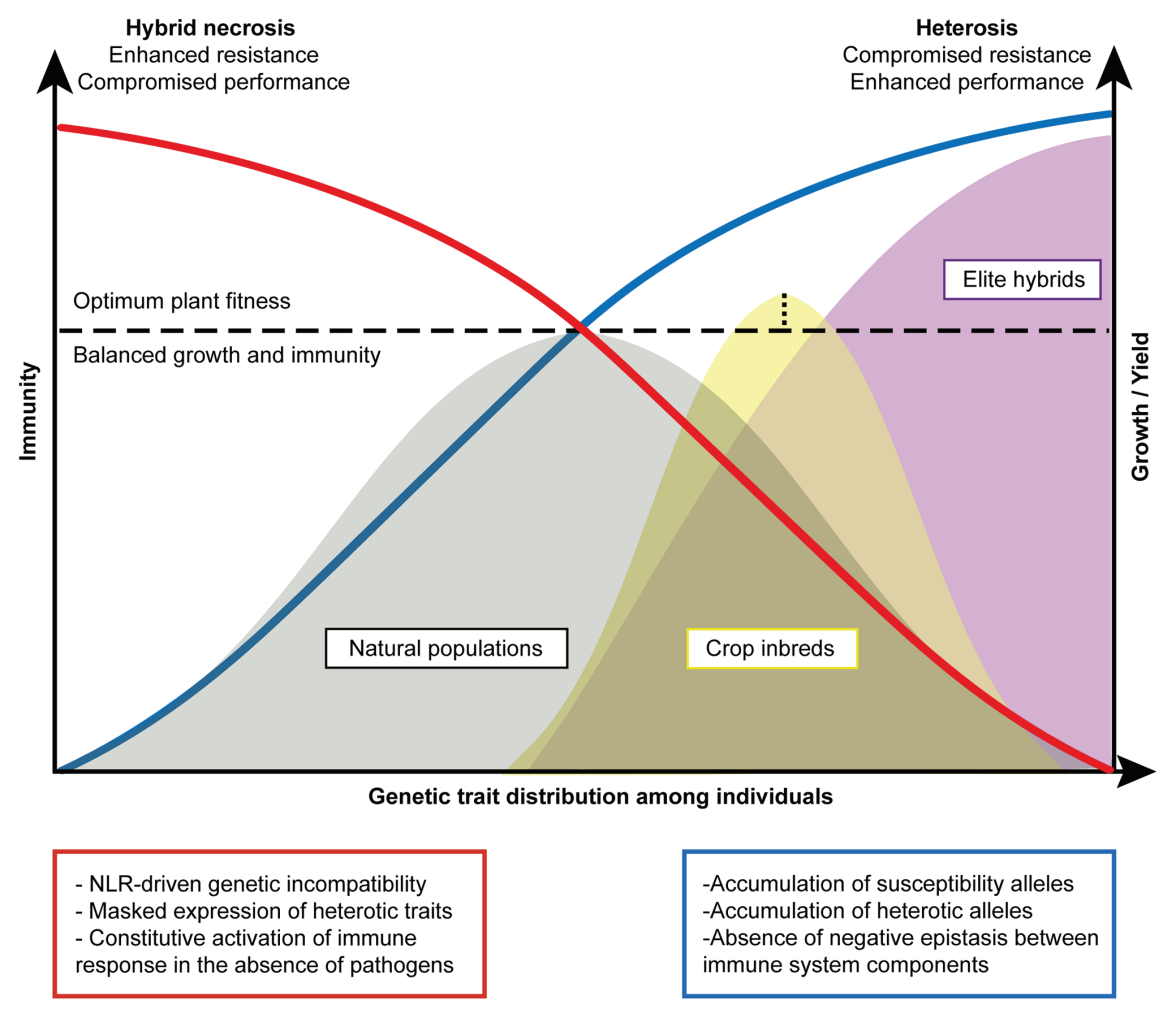
Phenotypic expression of hybrid necrosis vs. heterosis. The model represents an equilibrium between immunity and growth and/or yield achieved essentially to ensure optimum plant fitness (horizontal dotted line). The expression of two extreme phenotypes, heterosis (blue) and hybrid necrosis (red), results from drastic disruption of the equilibrium. The optimum fitness equilibrium is predominantly reached by natural populations (gray area) with robust immune systems characterized by a broad and controlled immune response to biotic stresses. The total frequency of resistant alleles in the natural population potential limits overall growth and yield as compared to commercial lines. Crop inbred lines (yellow area) show a disproportioned accumulation of heterotic alleles. In optimal growth conditions, the fitness of these lines may be superior to the one reached by natural populations (vertical dotted line). Predominant accumulation of heterotic traits shifts the distribution of these lines toward the right, prioritizing the expression of heterosis over a robust immune system. Elite hybrids (purple area) show the highest accumulation of heterotic alleles accompanied by the accumulation of susceptibility alleles for blast rice and leaf-blight (Huang et al., 2015). The performance for elite hybrids can surpass the optimum fitness point under controlled conditions. Nonetheless, in three scenarios, the expression of heterosis may be compromised being: (i) hybrid necrosis, (ii) introduction of *R* genes to the cost of yield, and (iii) susceptibility to pathogen attack.

Here, we illustrate an equilibrium model that represents different manifestations of the trade-off between growth and immunity in natural populations, crop inbred lines, and elite hybrids on the basis of genetic trait distribution for disease resistance and heterosis (Figure 2). We propose that hybrid necrosis and heterosis lie in the extreme end of such equilibrium as the result of disproportionate phenotypic manifestations of uncontrolled immunity and exacerbated growth, respectively. As explained above, the genetic architecture regarding the *R* repertoire affects plant fitness both in the presence and absence of pathogens. In addition, the accumulation of heterotic alleles corresponds to an increase in growth and yield, as long as they contain an optimum *R* repertoire that does not cause yield penalty. In a given natural population (gray) where multiple types of immune alleles are being maintained, we envision that the fitness level of individuals in the population would show a wide distribution with a peak that reaches the point optimally balanced with the two opposing forces, immunity and growth (Figure 2). Under a pathogen attack, immune responses in the natural population are activated only among the individuals carrying the cognate immune receptors that can mount robust immunity. On the other hand, non-carriers would suffer serious disease symptoms which eventually decrease the allele frequency of the susceptibility alleles in the population. However, when the pathogenic load is low or absent, the discrepancy in allele frequencies between the carriers and non-carriers becomes rebalanced because the fitness cost in the carriers would render them as unfavorable ones over the non-carriers (Tian et al., 2003; Karasov et al., 2014). For instance, stable natural populations of *A. thaliana* would experience bouts of such fluctuating events given that pathogenic loads in nature are believed to be stochastic (Karasov et al., 2018). In our model, as the immunity and growth traits are simplified to manifest as antagonistic forces, the high genetic diversity in the immune system will result in high diversity in growth traits. Thus, compared to selectively bred materials, natural populations would show the widest genetic trait distribution (gray; Figure 2). On the other hand, in the inbreeding crop parents (yellow) that are developed to generate elite hybrids, the peak of equilibrium shifts toward favoring growth as selective breeding has accumulated heterosis alleles (Figure 2). Some inbred lines could even reach a point exceeding the fitness equilibrium, resulting in enhanced resistance for certain pathogens yet with a minimized yield penalty. This has been achieved through domestication efforts that had largely reconstructed *R* gene repertoires in the genomes (Stein et al., 2018) and through breeding strategies successfully pyramiding a few *R* genes with the least yield penalty (Xiao et al., 2017; Chen et al., 2020). Under the breeding goal of enhancing yield and resistance in hybrids, *R* genes in inbred lines predisposed to show extreme deleterious phenotypes in later generations (i.e., hybrid necrosis) should have been eliminated or largely reduced. Additionally, a common breeding practice had favorably used only a small number of *R* genes which show broad and/or durable resistance for dominant field pathogens (Li et al., 2019). Thus, inbred individuals would show a much narrower distribution for resistant and heterotic traits as compared to ones found in natural populations (Figure 2). Hybridization can easily disrupt the fitness equilibrium to a large extent due to the presence of novel allelic combinations and genetic interactions (Figure 1) that often leads to antagonistic events: hybrid necrosis (red) or heterosis (blue; Figure 2). Elite hybrids (purple) would occupy space in this equilibrium reaching toward far right with the expression of maximum heterotic traits and of least number of resistance traits only suited for a purpose. Maximization of heterotic traits has been shown to be achieved due to the concomitant accumulation of heterotic and/or susceptible alleles in rice breeding (Huang et al., 2015, 2016; Lin et al., 2020). Therefore, this group is skewed toward increased growth and yield reaching a much higher level for optimum fitness if we consider that those plants are grown under controlled environments in the absence of pathogens or in the presence of only a known pathogen that can be resisted (Figure 2). Looking at the bigger picture, however, losses in rice production worldwide are certainly caused by a generalized compromise in immunity in elite hybrids, as no one can predict the next wave of pathogenic attack. We then propose that elite hybrids display an underperforming *R* gene repertoire with insufficient diversity to face a wide range of pathogenic strains. Such underrepresentation of optimum *R* repertoires in elite hybrids can be explained by the trade-off favoring growth and selection against hybrid necrosis alleles that might have great resistance potential.

## Molecular Mechanisms Underlying Hybrid Necrosis

One of the breeding goals in crops lies in broadening the spectrum of disease resistance. Understanding how plants have evolved the immune system to defend multiple pathogens is the first step to deploy different disease resistance traits into breeding panels. Although phytopathogens utilize diverse infection strategies, plants employ seemingly common yet complex immune pathways for defense (Mukhtar et al., 2011; Dou and Zhou, 2012; Weßling et al., 2014; Zhou and Zhang, 2020). The dedicated immune network is featured with prominent classes of immune receptors that perceive invasive events and transduce immune signaling. Coevolution between plants and pathogens drives the diversification of plant immune networks that immune receptors participate in; the system found in a given plant individual is supposedly optimized for its own, but not always function properly when combined in hybrids (Bomblies and Weigel, 2007; Chae et al., 2016). Hybrid necrosis is an extreme outcome of the immune system dysfunction resulting from newly combined immune components independently evolved from each parental lineage. In this section, we summarize recent advances that revealed the mechanism of plant immune receptor action and explain how such mechanistic understanding rationalizes the action of NLR variants predisposed for hybrid necrosis. From the molecular framework, we propose how uncontrolled autoimmune signaling could be derived due to the combination of incompatible immune components involving NLRs. Our mechanistic view on the NLR-based autoimmunity provides a strategy to optimize the growth-immunity equilibrium toward breeding elite hybrids.

### Diversity of NLRs in Sequence and Function

Faced with constant threats from pathogens, plants use a suit of membrane-bound and intracellular receptors to initiate defense signaling pathways to confer disease resistance (Kourelis and van der Hoorn, 2018). Most cell surface receptors are pattern-recognition receptors (PRRs), which include RLKs and RLPs. RLKs contain an extracellular LRR domain, a single-pass transmembrane (TM) domain, and an intracellular kinase domain. RLPs are structurally similar to RLKs except they do not contain a kinase domain (Boutrot and Zipfel, 2017). They could perceive the conserved non-self molecules, termed as pathogen-associated molecular patterns (PAMPs) or self-molecule damage-associated molecular patterns (DAMPs), and activate PAMP-triggered immunity (PTI; Boller and Felix, 2009; Boutrot and Zipfel, 2017). Some pathogens could yet evade from the PTI-based host immunity by secreting effectors that promote pathogenic virulence by suppressing immune perception or modifying host physiology (Toruño et al., 2016). NLRs are the family of immune receptors that sense the effectors or effector-driven modifications in the plant cell to initiate an acute immune response, ETI. Reflecting the effectiveness of ETI, NLRs constitute a major fraction of R proteins discovered to date across plant species (Cui et al., 2015; Kourelis and van der Hoorn, 2018). Depending on whether an effector and the matching NLR physically associate, the NLR recognition strategy could be divided into “direct” and “indirect” modes. The indirect recognition mode could be further refined to engage a third protein that can be classified either as “guard” or “decoy” in the plant cell (Van der Biezen and Jones, 1998; Dangl and Jones, 2001; van der Hoorn and Kamoun, 2008; Cesari, 2018). Homeostasis in the plant cell is gauged by the status of such host proteins, for which the matching NLRs monitor. Those host proteins become effector-targets upon infection, and NLRs sense such events to trigger ETI. Here, decoys are considered to have been generated to mimic guardees that have important immune functions. Under arms race between rapidly evolving pathogen effectors and host proteins, plants seem to have widened the net to catch the effectors by duplicating effector targets as decoys that in essence have no other roles affecting plant fitness (van der Hoorn and Kamoun, 2008; Ilyas et al., 2015).

NLRs are multidomain proteins with high diversity in structure and sequence. Based on the N-terminal structure, plant NLRs could be divided into three groups; substantial numbers of NLRs, in particular in eudicots, contain the Toll/Interleukin-1 receptor (TIR) domain at their N-terminus, termed as TIR-NLR or TNL in short; NLRs carrying predicted coiled-coil (CC) domains, albeit more variable than TIR in domain definition, are named CC-NLR or CNL; a less-abundant NLR group carries a non-canonical CC that bears similarity to RESISTANCE TO POWDERY MILDEW8 (RPW8) and named as RPW8-NLR or RNL (Shao et al., 2016). The small RPW8 proteins themselves serve as atypical non-NLR R proteins for conferring broad-spectrum resistance to powdery mildew, while some RPW8 domains experimentally cloned out from RNLs have been demonstrated to trigger cell death on its own when expressed in *Nicotiana benthamiana* (Xiao et al., 2001; Collier et al., 2011). Interestingly, one of the hybrid necrosis *DM* loci, *DM7*, encodes multiple members of RPW8s and HOMOLOG OF RPW8s (HRs), highlighting their connection to cell death activity (Barragan et al., 2019). The central part of NLRs is occupied by the nucleotide-binding and Apaf-1, R protein and CED4 homology (NB-ARC) domain followed by the C-terminal leucine-rich repeat (LRR) domain. The NB-ARC domain offers a conserved nucleotide-binding pocket, while LRR plays an essential role in effector recognition and features the highest sequence variability compared to other domains (Collier and Moffett, 2009; Takken and Goverse, 2012; Cesari, 2018; Wang et al., 2019a,b). The modular nature in domain structure, as well as high variability in sequence and copy number, are the key NLR features which presumably promote its versatility in the plant immune system.

**Figure 3:**
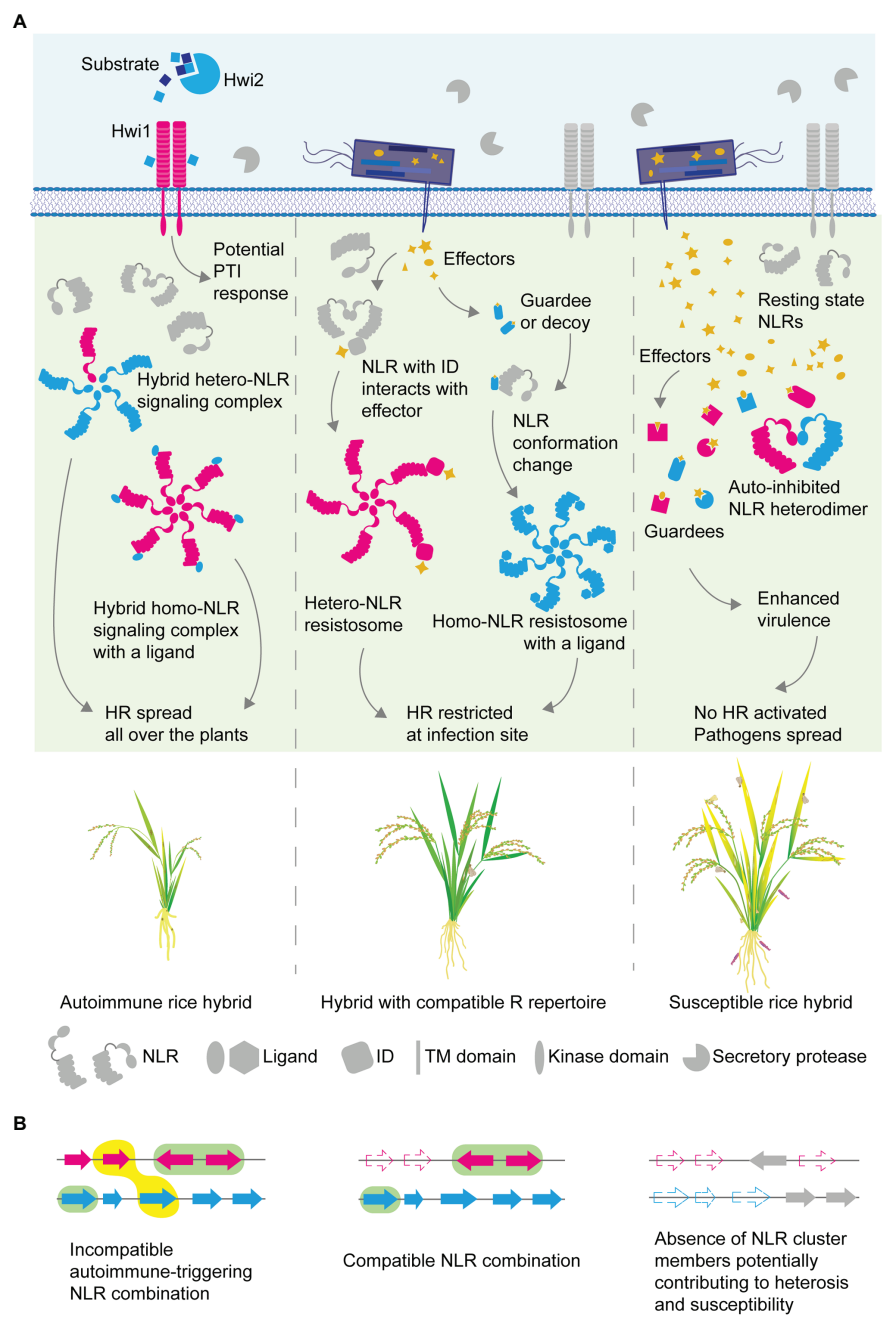
Molecular mechanisms for disease resistance, hybrid necrosis and heterosis in the view of NLR receptor complex formation. **(A)** NLR complex assembly illustrated in the plant cell of autoimmune (left), normal (middle) or heterotic (right) hybrids. Immune components are color-coded as magenta or cyan to indicate parental origins. Different architecture in NLR illustration, such as a fusion of ID at the C-terminal end, was used to differentiate distinct NLR proteins in the hetero-complex. Domain illustrations constituting the membrane-bound receptor-like kinase (RLK)/receptor-like proteins (RLPs) and intracellular NLRs are indicated at the bottom. The resting state is indicated with gray fill while the active state is with cyan or magenta. Effectors are filled with orange. In a rice hybrid with compatible immune components (middle), effector-modified host proteins are perceived by NLRs, which induces the assembly of a signaling complex, so-called resistosome. NLR-IDs with a decoy domain are hypothesized to form a resistosome with their pair NLRs, forming a heteromeric complex (middle). Conformation changes occurring upon perception are indicated with the closed-form of NLRs and linear form of NLRs. In an autoimmune hybrid (left), signaling NLR complexes form constitutively without effector perception. The complex may consist of NLRs of different parental origins, or of NLR and other host proteins that function as ligands to activate the NLR to initiate and complete the assembly. RLKs of one parent could be constitutively activated by ligands from the other parent to trigger pathogen-associated molecular pattern (PAMP)-triggered immunity (PTI). Accumulation of susceptibility alleles or interference between NLRs in breeding lines (right) can lead to hybrids with compromised resistance. NLR underrepresentation may facilitate effector action toward disease. **(B)** Schematic diagram of NLR cluster composition assembled in hybrids. The incompatible NLR combination (left) is highlighted with a yellow-filled object. Compatible combinations are marked with green-filled objects (left, middle). Each filled arrow indicates an NLR, while pseudogenization or locus deletion is represented with dashed-lined arrows. Arrows are colored to indicate different parental origins.

Beyond a classical role of NLRs conferring a strain-specific disease resistance, recent findings highlight functional diversity of NLRs in the plant immune system. It is apparent that some NLRs form a pair, commonly encoded in head-to-head orientation in the genome, and require each other to fulfill resistance functions (Sinapidou et al., 2004; Narusaka et al., 2009; Okuyama et al., 2011; van Wersch and Li, 2019). Another group of NLRs assists immune signaling initiated from other NLRs, which are named as helpers. Well-characterized helpers in *N. benthamiana* and *A. thaliana* belong to RNLs (Peart et al., 2005; Bonardi et al., 2011; Collier et al., 2011; Castel et al., 2019), while helpers found in multiple Solanaceae species belong to a clade of CNL (Wu et al., 2016, 2017). Diversity in NLR function is likely to reflect the evolutionary processes followed by the massive expansion of NLRs in the plant lineage. Indeed, a recent pan-NLRome analysis carried out in *A. thaliana* has shown that distinct patterns of sequence polymorphisms exist for the different functional NLR groups (Van de Weyer et al., 2019). Paired NLRs evolve as one functional unit, while classical NLRs conferring resistance to adapted pathogens show the highest sequence diversity in populations, indicating diversifying forces imposed on such NLRs. On the other hand, helpers remain relatively conserved, meeting the needs to work with multiple sensors. A surprising versatility of NLR is also found in its modularity incorporating extra domains into its classical tripartite domains. In particular, domains found in decoys are integrated as part of NLRs as a single translation unit. This type of NLR is called NLR-ID with ID standing for the “integrated decoy” (Cesari et al., 2014a; Wu et al., 2015). Decoy domains of NLR-IDs are known to readily sense the effector presence through physical association, and thus NLR-IDs are considered as sensor NLRs (Cesari, 2018; Adachi et al., 2019). The concept of the sensor has been elaborated through the identification of functionally dependent paired NLRs in rice. Rice NLRs, the RGA4/RGA5 and Pi5-1/Pi5-2 pairs require each other to confer resistance to rice blast fungus *M. oryzae* through one NLR of the pair, the sensor, which directly binds with the matching effectors through its ID (Lee et al., 2009; Okuyama et al., 2011; Cesari et al., 2013, 2014b). Functional dependency is explained by their ability of forming a heterodimer, in which effector sensing through the ID in the sensor NLR instigates the other to activate ETI to constrain rice blast growth (Cesari et al., 2014b; Figure 3A, middle). In this partnership, the matching NLR to the sensor is considered as an executor NLR due to its ability to transduce immune signaling leading to the HR, of which action appears to be under a negative control of the partnering sensor in the absence of pathogenic effectors. Up to date, a handful of NLR pairs are found under the same working mechanism: in *A. thaliana* RPS4/RRS1, CHS3/CSA1 NLRs are characterized as pairs with one NLR as a sensor with ID and the other as an executor (Cesari et al., 2014b; Huh et al., 2017; Ma et al., 2018). Although the initially characterized NLR-IDs haven been discovered by their head-to-head configuration in the genomic loci, later systematic search for NLR-IDs across plant genomes revealed that such configuration is not always the case, implying that a gained functional dependency might have promoted a genomic relocation of non-paralogous NLRs to be placed in tandem under linkage to facilitate coevolution of the two.

Sometimes, NLRs residing within a cluster can impede the signaling capacity of the paralogous NLRs. One example is the *Pigm* multigene cluster in rice that has been utilized for conferring broad-spectrum resistance to *M. oryzae* in many regions in China over the decades of breeding (Deng et al., 2017). The enhanced *R* trait is conferred by *PigmR* encoding a CNL, while one of the closely related paralogs *PigmS* in the same cluster opposes the *PigmR*’s function in a tissue-specific manner. As being a strong *R*, *PigmR* shows clear yield penalty as its uncontrolled function in the absence of antagonizing *PigmS* could cause a reduction in grain weight and size, potentially through autoimmunity. It has been shown that PigmS and PigmR could form a heterocomplex through their CC domains and thus it is plausible that the heterocomplex formation might inhibit the formation of PigmR homo-signaling complexes required for strong immunity. In the extant high-yielding rice lines, the *PigmS* promoter is highly methylated and the expression is suppressed in foliar tissues but not in the pollen. The tissue-specific expression would condition the trade-off to be balanced differently. In the vegetative tissue, PigmR homo-complexes would form to confer broad-spectrum resistance to *M. oryzae* when infected or to exert some degree of yield penalty when not infected. However, the net yield penalty is much alleviated as the epigenetic repression of the yield-saving *PigmS* expression is released in the pollen: supposedly PigmR/PigmS heterocomplex would reduce *PigmR*-mediated detrimental effects in the pollen, such as cell death or residual autoimmunity, and healthy pollen improve grain quality. This is a great example of how epigenetic regulation, potentially selected by rice breeding, contributes to achieve a balance between resistance and yield (Cesari and Kroj, 2017; Deng et al., 2017). While antagonistic paralogs could positively regulate the trade-off through tissue specificity, it can also hinder efforts to pyramid paralogous NLRs. When an introgressed paralogous NLR find its partner in the recipient germplasms to form a non-functional heteromeric NLR complex, the desired immunity from the introgressed *R* traits would be suppressed (Figure 3A, right), of which molecular examples are reported in wheat breeding (Hurni et al., 2014; Stirnweis et al., 2014).

Contrary to NLRs requiring or assisting others for full functionality, there are NLRs self-sufficient to complete its function without the need of other NLRs (Adachi et al., 2019). ZAR1 in *A. thaliana* is the most well-characterized NLR of which structure is recently resolved to reveal successive events accompanying NLR activation and signaling complex formation (Wang et al., 2019a,b). The ZAR1 structure revealed by cryo-EM explains how the previously postulated “on” and “off” status influence NLR activity and signaling complex assembly. The resolved complex includes not only ZAR1 but also resistance-related kinase 1 (RKS1) bound to the LRR domain of ZAR1. When ADP is bound to the NB-ARC of ZAR1, the ZAR1-RKS1 complex remains at resting state and inactive. Yet, when the effector-driven uridylation on PBL2 occurs, RKS1 tethers it to form a ZAR1-RKS1-PBL2^UMP^ complex and ADP/dATP exchange occurs, which is a key process ensuing conformational changes of ZAR1 and successive oligomerization. The resulting full signaling ZAR1 (or ZAR1-RKS1-PBL2) complex appears as a wheel-like pentamer, which is referred as ZAR1 resistosome that is sufficient to fully trigger the HR to restrict *Xanthomonas campestris* bacterial growth (Wang et al., 2019a,b; Figure 3A, middle). The mechanism underlying the NLR assembly offers an excellent template to envision NLR action and (auto)immunity, in general, to explain how plant performance is affected by controlled or uncontrolled immunity in hybrid plants, which we discuss in the next section.

### Mechanisms of NLR-Mediated Autoimmunity Responsible for Hybrid Necrosis

Hybrid necrosis caused by immune system incompatibility appears to result from too much variability in the NLR sequences and potentially in function. Almost all NLRs predisposed to hybrid necrosis are encoded by tandem clusters (Figure 3B); a recently cloned *DM10* is encoded by a single locus, however, the locus had been generated through a recent chromosomal relocation event of a multigene TNL cluster in *A. thaliana* (Barragan et al., 2020). Moreover, the most prominent *DM2* cluster along with other large *DM* clusters show great expandability in cluster size with high CNV detected in the 64 natural accessions (Lee and Chae, 2020). An interesting finding from the pattern analysis of NLR cluster expansion is that not all NLRs in the cluster contribute to the expansion in the global *A. thaliana* population, suggesting that differentiated functionality within a given NLR cluster may contribute to shaping the NLR repertoire available in the population. Potentially deleterious hybrid necrosis NLR alleles may rise during cluster expansion and contraction, which in some clusters may not be easily purged but maintained due to the linkage to beneficial *R* alleles within the cluster.

The first cloned BDM pair for hybrid necrosis in *A. thaliana* is the two NLR-encoding genes, DM1 and DM2d, and these NLRs have been shown to form a heteromeric oligomerized complex in plants through their TIR domains (Tran et al., 2017). A recent molecular investigation on DM6/DM7 hybrid necrosis also has shown that DM6/RPP7 NLR oligomeric status is augmented by the presence of the matching hybrid necrosis pair DM7, RPW8/HR protein through physical interaction between the two proteins (Li et al., 2020). Based on the ZAR1 resistosome structure, we postulate that in the autoimmune hybrids the causal NLR pairs are likely to formulate a hetero-signaling complex consisting of NLRs from different parents. If the BDM pair involves a non-NLR protein and an NLR, the NLR complex would include the host protein such as in RPP7/RPW8-mediated autoimmunity, which is also reflective of the ZAR1 NLR complex including decoys (Figure 3A, left and middle). The hybrid NLR signaling complex exerts immune responses in an uncontrolled manner since the completion of the signaling complex would not depend on an effector-trigger but on their own conformation of participating NLRs. Naturally evolved NLR variants with high sequence similarity have been experimentally demonstrated to potentiate different degrees of signaling (Bernoux et al., 2016). Based on the comparative analysis of closely related flax NLR L6 and L7, Bernoux et al. (2016) proposed that NLRs are present both in active vs. inactive status under equilibrium, which becomes readily shifted toward the active state when the effector is bound. We hypothesize that naturally occurring hybrid necrosis NLR variants adopt conformations close to the active status in a way that the equilibrium is already pulled close to fully active status. Independently evolved NLRs from the reciprocal parent would mistakenly recognize this form as an effector-bound conformation and assemble a fully functional NLR signaling complex. This proposition reminds a situation of paired NLR, where one is an effector-bound sensor and the other is a signaling executer (Figure 3B, middle). Although we proposed that DM1 and DM2d fit under this molecular category, with DM2d masquadering an effector-bound conformation and DM1 recognizing this form to help signaling, none of them carries IDs (Tran et al., 2017). It would be interesting to uncover molecular details of more hybrid necrosis cases, which will expand our knowledge on the functional diversity of NLRs in complex assembly and signaling. The new findings on RPW8/HR4 facilitating a higher-order NLR assembly also reminds of the ZAR1 complex assembly initiated by decoy binding (Figure 3B, left and middle). Whether or not RPW8 proteins are effector targets and how this class of proteins potentiate cell death remain as an active area of research. Our proposition of anti-hybrid necrosis potentially explaining heterosis could be illustrated with the molecular scenarios in the view of NLR complex formation. In the heterotic hybrids, NLR repertoires inherited from parents are supposedly much reduced as a result of selective breeding and the chance of formulating a hybrid NLR complex or decoy-assisted assembly of NLR complex is low (Figures 3A,B, right). Even worse scenario would be the auto-inhibiting NLR complex formation, which can suppress the particular disease resistance traits bred in the respective parents as was discussed in the above section with examples in wheat breeding (Hurni et al., 2014; Stirnweis et al., 2014).

In rice, most hybrid dysfunction has been categorized under hybrid weakness. The case involving *Hwi1/Hwi2* caught our attention as *Hwi1* encodes two tandemly repeated RLKs from wild rice and *Hwi2* encodes a secreted putative subtilisin-like protease in the *indica* rice variety Teqing (Chen et al., 2013, 2014). With the established contribution of RLKs to PTI, we could hypothesize that Hwi1 from wild origin mistakenly perceives Hwi2-generated products as PAMPs or DAMPs in the hybrid, presumably activating uncontrolled PTI at the cost of yield in the hybrid rice (Figure 3A, upper left). Another immune-related factor contributing to hybrid performance in rice is S5, a disease resistance-related aspartic protease (AP). Allelic conflict at S5 contributes to set up a reproductive barrier between *indica* and *japonica* by interfering with the functional embryo-sac production (Chen et al., 2008; Ji et al., 2012; Yang et al., 2012). Although the AP family has not yet been investigated in line with NLR activity, members of this family are involved in defense responses with activated SA in addition to pollen and ovule development in multiple plant species, including rice, grape and *A. thaliana* (Xia et al., 2004; Ge et al., 2005; Prasad et al., 2009; Huang et al., 2013; Guo et al., 2016; Gao et al., 2017). Given that immune incompatibility between the protease Rcr3 from domesticated tomatoes and R protein Cf-2 from wild tomatoes are known, it will be interesting to see if the rice AP important for setting up the reproductive barrier would engage an immune system incompatibility under BDM epistasis. Breeding history and already existing *indica-japonica* rice hybrids that overcame the AP-mediated barrier would provide a hint on the involvement of immune components as well as the possibility of APs as host targets that an NLR monitors.

## Reverse Breeding Application to Hybrid Necrosis and Heterosis

**Figure 4:**
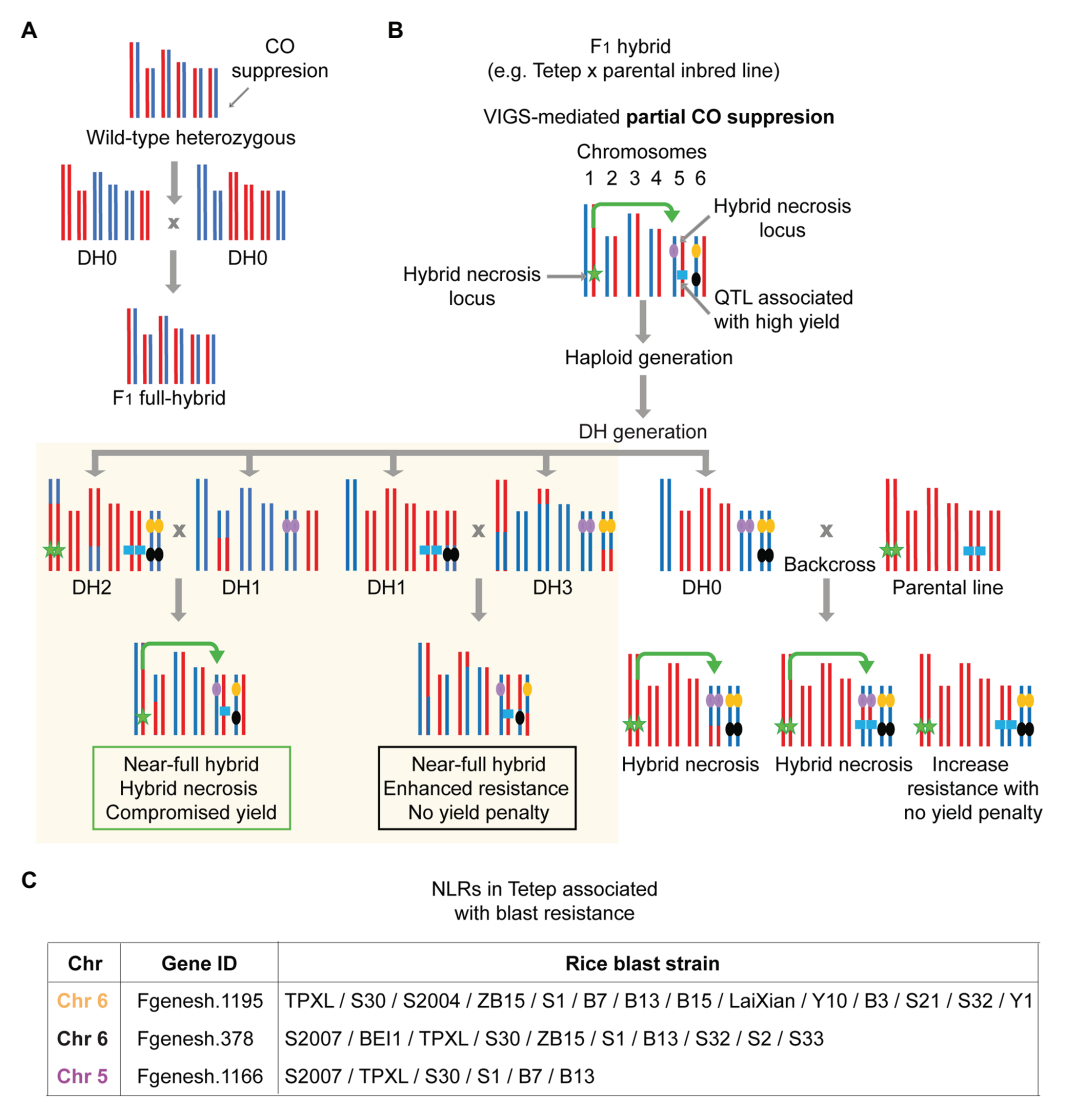
Reverse breeding through virus-induced gene silencing (VIGS)-mediated partial crossover suppression in a rice hybrid. **(A)** Reverse breeding allows the recreation of a heterozygous plant as an F_1_ hybrid through the generation of genetic complementing parental lines. **(B)** A schematic reverse breeding strategy in rice. Only the first six chromosomes of a putative rice hybrid between Tetep (blue) and a hypothetical parental inbred line (red) are presented for simplicity. Three NLRs found in the Tetep genome are represented with purple, yellow, and black circles in chromosomes 5 and 6. The quantitative trait locus (QTL) associated with high yield is represented by the light blue square in chromosome 5. Hypothetical genetic interaction between two loci (green star and purple circle) results in hybrid necrosis, thus affecting yield. Reverse breeding based on partial suppression of meiotic recombination will yield plants producing gametes carrying chromosomes with 0, 1, 2, or 3 crossover (COs) which will be grown into DH0, DH1, DH2, and DH3, respectively. Different DHs can be crossed to produce near-full hybrids. The homozygous regions found in one near-full hybrid eliminate one of the hybrid necrosis loci (green star) while retaining the QTL for increased yield and three NLRs from Tetep, hence allowing the generation of a resistant and high-yielding hybrid. The DH0 lines [chromosome-substitution lines (CSLs)] can be used as parental donors to introduce *R* genes in a recipient background. Note that the lines obtained in this scheme can be instrumental to map hybrid necrosis loci **(C)**. List of NLRs in the Tetep represented in **(B)** and the list of pathogens they confer resistance against (Wang et al., 2019c). The font color corresponds to the NLR represented in each chromosome in **(B)**.

To improve the combining ability of antagonistic traits such as growth and immunity, understanding genetic architectures underlying heterosis and hybrid necrosis is the first step. With the advances in crop genomics in recent years, it is becoming feasible to map responsible loci associated with such traits and retrieve relevant SNP sets, which will guide the design of a crop based on genomic information. However, challenges still remain to precisely and efficiently incorporate new traits into elite varieties. Given that fine-mapping resolution in crops still span a region including multiple loci and that immune genes particularly tend to form a cluster in the genome, substituting a responsible chromosome segment can be a useful genetic solution both to detect causal loci in bulk and to incorporate allele replacement when causal genes had not been cloned (Balakrishnan et al., 2019). Reverse breeding is an effective way to fix heterosis from hybrids expressing a suit of beneficial traits (Dirks et al., 2009). It first decomposes traits by generating numerous homozygous lines from a given heterozygous plant, from which desirable phenotypic traits can be selected in combination or in part. The crossing of complementing homozygous lines for the desired traits yields a hybrid which resembles fully or partially the genome of the starting heterozygous (Wijnker et al., 2012; Calvo-Baltanás et al., 2020; Figure 4A).

The technical application of reverse breeding demands two conditions: (1) the downregulation of meiotic recombination to preserve the two parental haplotypes that were combined in the starting hybrid and (2) the regeneration of the resulting gametes as a double-haploid (hereafter DH) population to obtain homozygous lines (Dirks et al., 2009; Wijnker et al., 2012; Figure 4). To date, reverse breeding has been shown in the model organism *A. thaliana* through complete and partial crossover (hereafter CO) suppression by silencing genes essential for meiotic recombination (Wijnker et al., 2012; Calvo-Baltanás et al., 2020). In the latest design, a meiotic gene required for about 83–87% of the total number of COs, *MSH5* (*MUT-S HOMOLOG 5*; Higgins et al., 2004, 2008; Lu et al., 2008), was silenced in a wild-type hybrid using virus-induced gene silencing (VIGS; Calvo-Baltanás et al., 2020). The silencing of *MSH5* resulted in non-recombinant and low-recombinant chromosomes segregating to gametes. These gametes are later grown into DH lines which show variable recombination rates, from zero COs (DH0) to three COs (thus generating DH1, DH2, or DH3; Figure 4B). If gametes carrying non-recombinant chromosomes are grown into DHs (DH0), chromosome-substitution lines (CSLs) will be obtained. The combination of two fully complementing CSLs will reconstitute the initial heterozygous genotype (Figure 4A). Gametes carrying low-recombinant chromosomes, however, can also be of use for reverse breeding. The combination of DH1 and DH2 lines, with one and two CO, respectively, will generate near-full hybrids (Figure 4B). Near-full hybrids largely retain the heterozygosity of the full hybrid with the exception of certain homozygous regions (Figure 4B). These hybrids, therefore, can be used to experimentally assess the effect that such homozygous regions have on hybrid performance (Calvo-Baltanás et al., 2020).

Pyramiding *R* genes in elite-hybrids is challenging, especially when it entails *R* gene cloning, which is particularly complicated due to massive structural variation often associated with the encoding loci (Jiao and Schneeberger, 2020). Alternatively, a backcrossing scheme between a donor and recipient line can facilitate the introduction of *R* genes in breeding lines. However, this is a time-consuming process and unknown deleterious combinations can lead to hybrid necrosis when parents containing newly assembled *R* genes sets are used to produce hybrids. To increase the efficiency of this process, one can use reverse breeding as an alternative technique to NLR cloning or to assist in backcrossing schemes (Figure 4B). In an ideal scenario, the genome of the initial (wild-type) heterozygote for a reverse breeding experiment will contain loci associated with hybrid necrosis, heterosis, and resistance. Reverse breeding based on partial CO suppression applied to this heterozygote will deliver DHs that will segregate for the desired number of *R* genes, heterotic loci and hybrid necrosis loci (Figure 4B). The combination of these lines will then produce hybrids with different genetic composition. Because DHs are grown from gametes that contain recombinant and non-recombinant chromosomes, depending on the combination of DHs used, some regions will become homozygous in the near-full hybrids (Figure 4B). In the event that such homozygous regions are not associated with yield, the expression of this trait should not be compromised in near-full hybrids. Some works already showed that the accumulation of several positive alleles was responsible for the heterotic traits in rice and for this reason, the reconstitution of the full hybrid genotype would not be essential to obtain elite heterozygous lines from a reverse breeding experiment (Huang et al., 2015, 2016). In addition, homozygous regions present in the near-full hybrids may be necessary to eliminate hybrid necrosis loci while retaining all the other desirable traits (i.e., *R* genes and heterotic loci; Figure 4B).

The use of reverse breeding offspring has further advantages. DH0s are also CSLs, a type of mapping population useful to dissect epistasis (Nadeau et al., 2000; Spiezio et al., 2012; Ziolkowski et al., 2017; Wijnen et al., 2018). CSLs can also be used as donor lines for single or entire sets of *R* genes; crossing CSLs to an existing breeding line allows the sequential introduction of desirable *R* genes into that background (Figure 4B). Because only specific chromosomes segregate, the associated genotyping effort to identify the lines with the desired introgression is largely reduced as compared to traditional backcrossing schemes. Moreover, if CSLs are crossed to an inbred parental line, in later generations, individuals segregating for specific loci will be obtained (Figure 4B). Such lines may contain a single chromosome segment from a donor parent introduced to a second, recurrent background. These genetic stocks are valuable tools for crop improvement, as they allow to map QTLs, causal genes, and gene interactions (Balakrishnan et al., 2019). Therefore, the rapid generation of CSLs through reverse breeding could be instrumental to facilitate the study of the genetic mechanisms of hybrid necrosis in rice.

### Practical Application of Reverse Breeding in Rice

For the application of reverse breeding in rice, silencing of genes essential for CO formation shall be achieved in hybrids. To this end, different approaches such as microRNA, RNAi, or VIGS can be used. There are, however, benefits of using VIGS over other silencing methods that require stable transformation. First, for a gene to be silenced by RNAi or microRNAs in the hybrid, at least one of the parental lines needs to be previously transformed with dominantly acting transgenes. Consequently, inbred lines for that particular hybrid need to be available. VIGS, however, allows direct silencing of the target in the chosen hybrid, even if this is a heterozygote from outcrossing populations. Therefore, VIGS represents a convenient, broad, rapid method over the use of stable transgenes. In rice, the rice tungro bacilliform virus (RTBV)-VIGS system has been shown to reduce gene expression by 80% (Kant and Dasgupta, 2017). The silencing efficiency of meiotic genes, however, is yet to be assessed using this system. The most obvious target for VIGS in a reverse breeding experiment would be *OsMSH5/OsMSH4* and/or other proteins involved in class I CO formation, which would lead to a comparable decrease in CO numbers as for the *A. thaliana* mutants defective in this CO pathway (Luo et al., 2013; Zhang et al., 2014, 2019; Wang et al., 2016; Calvo-Baltanás et al., 2020).

The effect of CO reduction on gamete viability can be calculated as (1/2)*^x^*, where “*x*” represents the species’ chromosome number. In rice, the average number of COs in wild-type meiosis has been estimated to be between 20.1 and 33.9 (Si et al., 2015; Zhang et al., 2019). The estimated 83% reduction in CO formation when class I CO formation is suppressed and considering 33.9 or 20.1 COs would result in about six or three residual COs per meiosis, respectively. While complete CO suppression would yield (1/2)^12^ × 100% = 0.02% of viable gametes, in the event that either six or three residual COs are present, and assuming the occurrence of one CO per chromosome pair this frequency will vary greatly, which will equal to (1/2)^12–6^ × 100% = 1.6% or (1/2)^12–3^ × 100% = 0.2%. Therefore, partial COs suppression represents an 80 to 10-fold increase in gamete viability over complete CO suppression, depending on the residual number of COs present. In addition, it is important to consider the number of gametes produced by a single plant, as this will affect the number of viable gametes obtained from a reverse breeding experiment in rice. The number of pollen grains produced by rice plants is rather variable among varieties, and it is influenced by anther-length and environmental conditions such as temperature (Fukushima et al., 2017). Under optimal growth conditions, each anther may contain 1,000–2,000 pollen grains (Fukushima et al., 2017). If COs are reduced by 83% and six or three residual COs are present, each anther will produce between 16 and 32 or between 2 and 4 viable pollen grains, respectively. Such pollen viability rate *a priori* may seem low, but it would certainly enable the recovery of reverse breeding offspring if several silenced anthers from each plant are used for DH regeneration.

The last step requires haploid and/or DH technology to be established. Although haploid-induction efficiency and DH regeneration in rice has long remained largely genotype-dependent, these constraints have been greatly overcome in recent years (Bishnoi et al., 2000; Maharani et al., 2020). Indeed, different anther culture techniques allow DH-recovery frequency that ranges from 30 to 60% (Mishra and Rao, 2016; Ren et al., 2017; Sarao and Gosal, 2018). Interestingly, one of the latest protocols available for anther-culture using an elite *indica* rice hybrid even reports a regeneration of exclusively DHs from callus (Naik et al., 2017). Such frequencies integrated in a reverse breeding scheme implies that useful DHs and near-full hybrids can be obtained in a maximum of two generations from the starting hybrid (Figure 4B).

## Conclusion

In this review, we put forward our proposition that hybrid necrosis resulting from immune incompatibility would function as a potential opposing force to the expression heterosis in hybrids. Our reasoning bases on recent molecular and genetic findings on hybrid necrosis in plant species covering both natural and selectively bred germplasms. Autoimmunity is a major mechanism underlying hybrid necrosis arising from a new combination of highly diversified immune components from different parental origins, which obligatorily results in compromise in growth and yield. Thus, hybrid necrosis illustrates an extreme degree of trade-off manifestation between growth and immunity. In the plant performance equilibrium model that we propose, the shifted equilibrium to the extremes is expressed as either hybrid necrosis or heterosis. Although the degree of contribution made from anti-hybrid necrosis to determining heterosis is yet to be examined, yield penalty associated with enhanced resistance observed throughout breeding history, including hybrid breeding in rice, clearly points to the link between heterosis and disease resistance traits. We encourage rice researchers to revisit the cases of underperforming hybrids in the breeding panel under the new concept of hybrid necrosis as an opposing force to heterosis. Despite the seemingly low breeding interests, such underperformance, when addressed under epistatic interactions and environmental variables affecting performances, might reveal an underestimated contribution of hybrid necrosis to restrict the full manifestation of heterosis. The findings will not only shed light on guided breeding strategies in the post-genome era but also greatly inform us of evolutionary processes shaping up an immune system including the valuable *R* gene repertoire. The more we understand the downside of hybrid performance, the better could we combine beneficial traits of yield and disease resistance. Valuable rice germplasms bred throughout history await a new wave of immune-centered breeding for heterosis.

## Author Contributions

VCB, JW, and EC formulated concepts and wrote the manuscript. All authors contributed to the article and approved the submitted version.

### Conflict of Interest

The authors declare that the research was conducted in the absence of any commercial or financial relationships that could be construed as a potential conflict of interest.
